# Molecular monitoring of causative viruses in child acute respiratory infection in endemo-epidemic situations in Shanghai

**Published:** 2011-01-10

**Authors:** Wei Wang, Philippe Cavailler, Peijun Ren, Jing Zhang, Wei Dong, Huajie Yan, Sek Mardy, Johann Cailhol, Philippe Buchy, Jun Sheng, Arnaud Fontanet, Vincent Deubel

**Affiliations:** 1 Key Laboratory of Molecular Virology & Immunology, Institut Pasteur of Shanghai, Chinese Academy of Sciences, Unit of Emerging Viruses, Shanghai, PR China; 2 Unit of Virology, Institut Pasteur in Cambodia, Phnom Penh, Cambodia; 3 Shanghai Nanxiang Hospital, Pediatric Department, Jiading District, Shanghai, PR China; 4 Unit of Epidemiology of Emerging Diseases, Institut Pasteur, Paris, France

## Background

Numerous viruses are responsible for respiratory infections; however, both their distribution and genetic diversity, in a limited area and a population subgroup, have been studied only rarely during a sustained period of time.

## Methods

A 2-year surveillance program of children presenting with acute respiratory infections (ARIs) was carried out to characterize the viral etiology and to assess whether using gene amplification and sequencing could be a reliable approach to monitor virus introduction and spread in a population subgroup.

## Results

Using multiplex RT-PCR, 15 different respiratory viruses were detected within the 486 nasopharyngeal positive samples collected among 817 children aged <9 years old who presented with ARI during October 2006 to September 2008. A single virus was detected in 373 patients (45.7%), and two to four viruses in 113 patients (13.8%). The most frequent causative viruses were respiratory syncytial virus (RSV) (24.7%), human bocavirus (24.5%), and human rhinovirus (HRV) (15%). RSV was more prevalent in winter and among young infants. Cases of seasonal influenza A and B viruses were reported mainly in January and August. An increase in adenovirus infection was observed during the spring of the second year of the study. Sequence analyses showed multiple introductions of different virus subtypes and identified a high prevalence of the newly defined HRV-C species. A higher viral incidence was observed during the winter of 2008, which was unusually cold.

## Conclusion

This study supports the usefulness of multiplex RT-PCR for virus detection and co-infection, and for implementation of a molecular monitoring system for endemic and epidemic viral respiratory infections.

